# Opioid Actions in Primary-Afferent Fibers—Involvement in Analgesia and Anesthesia

**DOI:** 10.3390/ph4020343

**Published:** 2011-01-28

**Authors:** Eiichi Kumamoto, Kotaro Mizuta, Tsugumi Fujita

**Affiliations:** Department of Physiology, Saga Medical School, 5-1-1 Nabeshima, Saga 849-8501, Japan; E-Mails: 08624027@edu.cc.saga-u.ac.jp (K.M.); fujitat@cc.saga-u.ac.jp (T.F.)

**Keywords:** opioids, spinal dorsal horn, excitatory synaptic transmission, primary-afferent fiber, action potential conduction

## Abstract

Opioids inhibit glutamatergic excitatory transmission from the periphery by activating G-protein coupled opioid receptors in the central terminals of primary-afferent neurons in the spinal substantia gelatinosa, resulting in antinociception. Opioid receptor activation in the peripheral terminals of primary-afferent neurons inhibits the production of action potentials in response to nociceptive stimuli given to the periphery, leading to antinociception. Opioids also exhibit a local anesthetic effect without opioid receptor activation in peripheral nerve fibers. This review article will focus on analgesia and anesthesia produced by the actions of opioids on primary-afferent fibers.

## 1. Introduction

**Figure 1:**
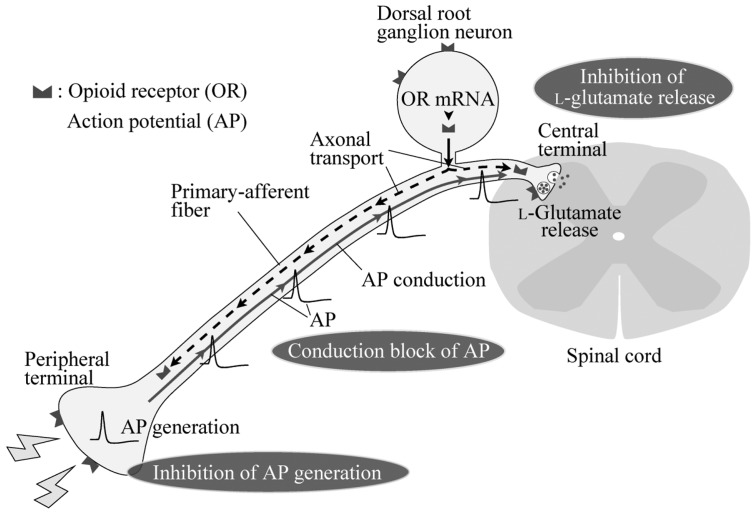
Schematic diagram illustrating the actions of opioids on dorsal root ganglion (DRG; primary-afferent) neurons. Opioids are known to inhibit the release of l-glutamate to spinal dorsal horn neurons from the central terminals of primary afferents and the generation of action potentials (APs) in the peripheral terminals of primary afferents, both of which actions are mediated by opioid receptors (ORs) which are synthesized in the somata of DRG neurons and transferred to the terminals by axonal transport. Opioids are also known to inhibit the conduction of APs in primary-afferent fibers without OR activation.

Nociceptive stimuli given to the skin produce an action potential (AP) in the peripheral terminals of primary-afferent neurons. Such an AP conducts through fine myelinated Aδ and unmyelinated C primary-afferent glutamatergic fibers contained in the dorsal root to the superficial laminae of the dorsal horn, especially the substantia gelatinosa (SG; lamina II of Rexed; [1,2]; Figure 1). This nociceptive information flows to the thalamus through a connection with projection neurons in lamina I and deeper laminae of the spinal dorsal horn [3], and then to the primary sensory area of the cerebral cortex, eliciting nociceptive sensation. A neuronal circuitry in the SG is thought to play an important role in the modulation of nociceptive transmission by various endogenous substances including opioids [4]. SG neurons preferentially receive in a mono- or polysynaptic manner Aδ- and C-primary afferent fibers, which carry fast and slow nociceptive information, respectively [5]. Inhibitory neurotransmitters, glycine and GABA, as well as l-glutamate, are involved in synaptic transmission in SG neurons in a polysynaptic manner from primary-afferent fibers.

Intrathecal administration of opioids produces a powerful analgesia in rats [6] and humans [7,8]. This analgesic effect is thought to be mediated by three principal subtypes of G-protein coupled opioid receptors, μ-, δ- and κ-type, the activation of which inhibits voltage-gated Ca^2+^ channels, activates inwardly-rectifying K^+^ channels or inhibits adenylate cyclase through the activation of G proteins [9]. These opioid receptors have been found in the superficial dorsal horn, especially the SG, in rats [10-13] and in humans [14]. Radioligand-binding experiments in the rat spinal cord have demonstrated that the most prevalent type of opioid receptors in laminae I-II is μ-type (63% or more) with considerably fewer of δ- (23% or less) and κ-type (15% or less; [10,15,16]). A partial (by 40–70%) reduction in the number of opioid-binding sites has been observed in the dorsal horn after the disruption of primary afferents by mechanical (dorsal rhizotomy; [10,11,17]) or chemical (the pretreatment with a selective fine-afferent neurotoxin, capsaicin; [18]) methods, indicating the localization of opioid receptors to both nerve terminals and postsynaptic neurons [19].

It has been reported that μ- or δ-opioid receptor agonists presynaptically inhibit glutamatergic synaptic transmissions in CNS neurons including superficial spinal dorsal horn [20-22], spinal trigeminal nucleus [23] and midbrain periaqueductal gray (PAG) neurons [24]. Alternatively, opioids may open one or more K^+^ channels through the activation of each of μ-, δ- and κ-opioid receptors and thus hyperpolarize membranes, resulting in an inhibition of excitatory transmission in CNS neurons including rat spinal cord [20,25] and spinal trigeminal nucleus neurons [23,26,27].

Although administration of opioids into the nerve sheath has been reported to lead to pain relief [28], many of pain treatments by use of opioids are based on systemic administration of conventional centrally-penetrating opioids, resulting in their actions in the PNS and CNS, both of which contribute to analgesia [29]. It is possible that centrally-injected opioids act on not only the CNS, but also the PNS, because King *et al.* [30] have demonstrated that opioids are transported from the brain to periphery by P-glycoprotein. In support of an important role of opioids in the PNS, subcutaneously-applied *N*-methylmorphine, which was shown not to penetrate the blood brain barrier, exhibited antinociception in an acetic acid-writhing model in mice [31]. Shannon and Lutz [32] have demonstrated that subcutaneous administration of an opioid loperamide, which hardly penetrates into the brain, produces antinociception in the formalin test in rats. Such an action of opioids in the PNS appears to be mediated by opioid receptors in the peripheral terminals of primary-afferent neurons [31-37].

Conduction of APs in peripheral nerve fibers is generally blocked by opioids, although Yuge *et al.* [38] have reported that there is not any significant change in the amplitude of compound action potential (CAP) in the superficial radial nerve following perineural application of an opioid morphine in decerebrate cats. For instance, opioids such as fentanyl and sufentanil reduce the peak amplitudes of CAPs recorded from peripheral nerve fibers [39] and inhibit peripheral nerve AP conduction [40]. Such a CAP inhibition is also produced by a non-narcotic opioid tramadol, (1*RS*; 2*RS*)-2-[(dimethyl-amino)methyl]-1-(3-methoxyphenyl)-cyclohexanol hydrochloride [41]. Jurna and Grossmann [42] have reported that an inhibitory effect of morphine on CAPs in mammalian peripheral nerve fibers is antagonized by a nonspecific opioid-receptor antagonist naloxone, indicating an involvement of opioid receptors. Consistent with this idea, binding and immunohistochemical studies have demonstrated the presence of opioid receptors in mammalian peripheral nerve fibers [43-45]. Hunter and Frank [46] also have reported a naloxone-sensitive inhibition of CAPs produced by opioids in frog sciatic nerve fibers. On the contrary, there are reports showing that opioids reduce CAP peak amplitudes [39] and inhibit nerve conduction [40] in a manner insensitive to naloxone.

This review article will mention our data about the actions of opioids on the central terminals of primary-afferent neurons and on peripheral nerve fibers and also other investigators' results about their actions on the peripheral terminals of primary-afferent neurons.

## 2. Opioid Actions on the Central Terminals of Primary-Afferent Neurons

It is possible that an opioid-induced inhibition of pain transmission is due to a negative modulation of glutamatergic transmission in the central terminals of primary-afferent neurons in the SG. In support of this idea, opioids administrated into the SG in anesthetized cats inhibited an excitation of deeper dorsal horn neurons caused by noxious peripheral stimuli without a change in their responses to innocuous stimuli such as touch [47]. In order to know a role of opioids in the nociceptive transmission, we examined their effects on l-glutamate-mediated excitatory postsynaptic currents (EPSCs) by applying the whole-cell patch-clamp technique to SG neurons in spinal cord slices dissected from the adult rat.

### 2.1. Actions of Opioid-Receptor Agonists

**Figure 2:**
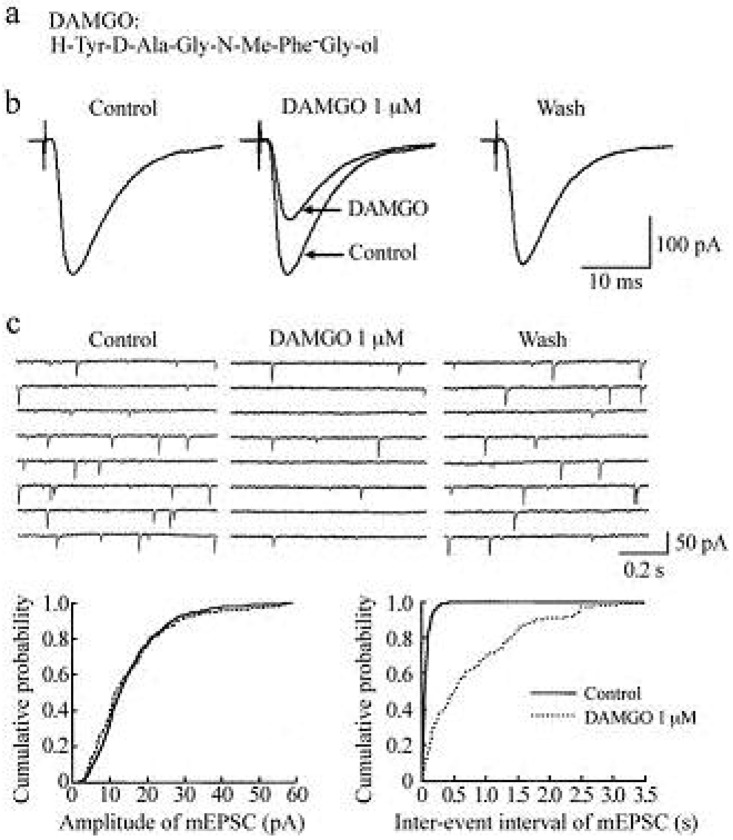
Actions of a μ-opioid receptor agonist DAMGO (1 μM) on glutamatergic excitatory transmission in rat substantia gelatinosa (SG) neurons. (**a**) The amino-acid sequence of DAMGO; (**b**) Averaged traces of six consecutive dorsal root-evoked monosynaptic Aδ-fiber excitatory postsynaptic currents (EPSCs) before (left), during (middle; where control EPSC in the left is superimposed for comparison), and after (right) the action of DAMGO. Note that DAMGO reversibly reduces the peak amplitude of the EPSC; (**c**) Upper part: eight consecutive traces of miniature EPSCs (mEPSCs) before (left), during (middle; 1 min after the beginning of DAMGO application), and after (right) the action of DAMGO; these were recorded in the presence of a Na^+^-channel blocker tetrodotoxin (TTX; 0.5 μM), a GABA_A_-receptor antagonist bicuculline (20 μM) and a glycine-receptor antagonist strychnine (2 μM). Lower part: cumulative distributions of the amplitude (left) and inter-event interval (right) of mEPSC, before (straight line) and under (dotted line) the action of DAMGO, which were obtained by analyzing 399 and 144 mEPSC events (which occurred during 60 and 120 s, respectively), respectively. DAMGO had no effect on the amplitude distribution (P > 0.5), but shifted the inter-event interval distribution toward a longer one (P < 0.01; Kolmogorov-Smirnov test). Data in the upper and lower parts were obtained from the same neuron. Note that DAMGO reduces the frequency of mEPSC without a change in amplitude. Holding potential (V_H_) used was −70 mV. Modified from [49].

**Figure 3:**
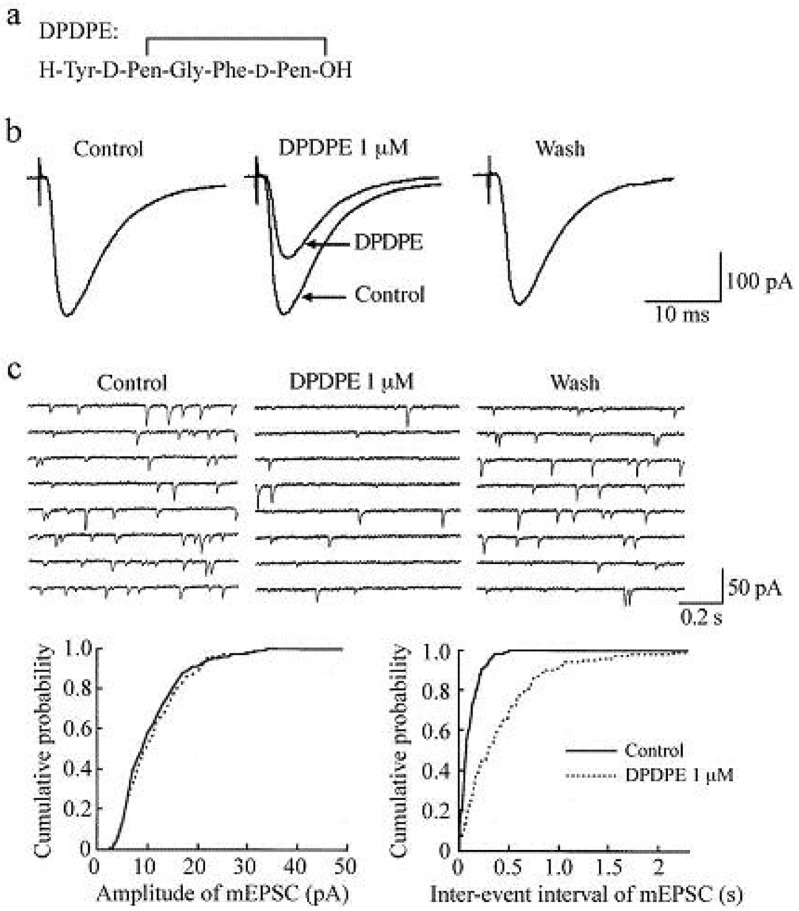
Actions of a δ-opioid receptor agonist DPDPE (1 μM) on glutamatergic excitatory transmission in rat SG neurons. (**a**) The amino-acid sequence of DPDPE; (**b**) Averaged traces of six consecutive dorsal root-evoked monosynaptic Aδ-fiber EPSCs before (left), during (middle; where control EPSC in the left is superimposed for comparison), and after (right) the action of DPDPE. Note that DPDPE as well as DAMGO reduces the peak amplitude of the EPSC; (**c**) Upper part: eight consecutive traces of mEPSCs before (left), during (middle; 1 min after the beginning of DPDPE application), and after (right) the action of DPDPE; these were recorded in the presence of TTX (0.5 μM), bicuculline (20 μM) and strychnine (2 μM). Lower part: cumulative distributions of the amplitude (left) and inter-event interval (right) of mEPSC, before (straight line) and under (dotted line) the action of DPDPE, which were obtained by analyzing 243 and 156 mEPSC events (which occurred for 60 and 120 s, respectively), respectively. DPDPE had no effect on the amplitude distribution (P > 0.7), but shifted the inter-event interval distribution toward a longer one (P < 0.01; Kolmogorov-Smirnov test). Data in the upper and lower parts were obtained from the same neuron. Note that DPDPE as well as DAMGO reduces the frequency of mEPSC without a change in amplitude. V_H_ = −70 mV. Modified from [49].

In the presence of a GABA_A_- and glycine-receptor antagonist, which are bicuculline (20 μM) and strychnine (2 μM), respectively, stimulation of a dorsal root with a low stimulus strength evoked a monosynaptic Aδ-fiber EPSC in SG neurons, as shown in Figure 2 and Figure 3. This Aδ-fiber evoked EPSC was completely blocked by 6-cyano-7-nitroquinoxaline-2,3-dione (CNQX; 20 μM), indicating an activation of non-*N*-methyl-d-aspartate (non-NMDA) receptors [48]. In 95% of neurons examined, superfusing a μ-opioid receptor agonist DAMGO (1 μM; Figure 2a) reversibly reduced the peak amplitude of the Aδ-fiber evoked EPSC, as shown in Figure 2b. The magnitude of this depression was 27%. In 71% of neurons tested, a δ-opioid receptor agonist DPDPE (1 μM; Figure 3a) also decreased the peak amplitude of Aδ-fiber evoked EPSC (Figure 3b) with an extent of 17%. When examined in neurons exhibiting inhibition of more than 5%, the DAMGO and DPDPE actions were not seen in the presence of a μ-opioid receptor antagonist CTAP (1 μM) and a δ-opioid receptor antagonist naltrindole (1 μM), respectively. Such a reduction in Aδ-fiber evoked EPSC amplitude was presynaptic in origin, because neither DAMGO (1 μM) nor DPDPE (1 μM) affected the peak amplitude of a response of SG neurons to a non-NMDA receptor agonist AMPA (10 μM). In contrast, a κ-opioid receptor agonist U-69593 (1 μM) had little effect on the Aδ-fiber evoked EPSC [49], although Randić *et al.* [50] have reported that κ agonists cause both potentiation and inhibition of excitatory transmission in SG neurons. These results indicate the presence of μ- and δ-type opioid receptors involved in inhibiting the release of l-glutamate from primary-afferent central terminals. A cellular mechanism for the presynaptic inhibition of evoked EPSC would be an inhibition of voltage-gated Ca^2+^ channels by opioids in nerve terminals, because opioids suppress Ca^2+^-channel currents in rat dorsal root ganglion (DRG) neurons [27,51]. DAMGO-sensitive adult rat DRG neurons seem not to express T-type Ca^2+^ (Cav 3.2) channels [52].

Although the central terminals of primary-afferent neurons express not only opioid receptors but also various types of neurotransmitter receptors, recent studies have revealed an interaction among the receptors in a synergistic (greater-than-additive) manner in inhibiting nociceptive transmission. For instance, Riedl *et al.* [53] have suggested that δ-opioid receptors may interact with α_2A_ adrenoceptors, the activation of which inhibits monosynaptic primary-afferent Aδ-fiber and C-fiber glutamatergic transmission in SG neurons [54].

Miniature EPSCs (mEPSCs) were isolated by adding a voltage-gated Na^+^-channel blocker tetrodotoxin (TTX; 0.5 μM) together with bicuculline (20 μM) and strychnine (2 μM) to superfusing Krebs solution. These were abolished by the addition of CNQX (20 μM), indicating an involvement of non-NMDA receptors [48,55]. The mEPSCs are produced by l-glutamate released onto SG neurons from the central terminals of primary-afferent neurons and glutamatergic interneuron terminals. In all neurons examined, superfusion of DAMGO (1 μM) resulted in a rapid and reversible reduction in the frequency of mEPSC without a change in the amplitude, as shown in the upper part of Figure 2c. The extent of this reduction was 61%. The lower part of Figure 2c demonstrates the action of DAMGO on cumulative distributions of the amplitude and inter-event interval of mEPSC.

While DAMGO increased a proportion of mEPSCs having a longer inter-event interval, it had no consistent effect on the cumulative distribution of mEPSC amplitude. In 75% of neurons tested, DPDPE (1 μM) also exhibited a similar action, as seen in Figure 3c; the frequency of mEPSC was reduced with an extent of 23% without a change in the amplitude. These effects on sEPSC frequency and amplitude indicate a decrease in the spontaneous release of l-glutamate from nerve terminals without a change in the sensitivity of non-NMDA receptors to l-glutamate. The DAMGO and DPDPE actions were not seen in the presence of CTAP and naltrindole (each 1 μM), respectively. On the contrary, U-69593 (1 μM) had little effect on the frequency of mEPSC [49]. These results indicate that μ- and δ-type opioid receptors also exist in glutamatergic interneuron terminals.

When examined at the same concentration of 1 μM, the sequence of efficacies of opioids in reducing either evoked EPSC amplitude or mEPSC frequency was μ- > δ- ≫ κ-type. This rank order was the same as that for the proportion of neurons in which each of the opioids exhibits evoked EPSC amplitude and mEPSC frequency reductions [49]. These results are in good agreement with that of analgesic effect caused by intrathecal injection of opioids in the rat, the potency order of which is μ- > δ- ≫ κ-type [56-58]. These appear to be due to a difference in density among the three types of opioid receptors expressed in the superficial dorsal horn (see above).

Recently, Zhou *et al.* [59] have reported that DAMGO (1 μM) produced not only a decrease in monosynaptic EPSC amplitude and mEPSC frequency but also a long-lasting increase in the amplitude and in the frequency in about a half of adult rat SG neurons examined. This heterosynaptic long-term potentiation has been attributed to opioid-induced hyperalgesia and tolerance.

### 2.2. Actions of Endomorphins

**Figure 4:**
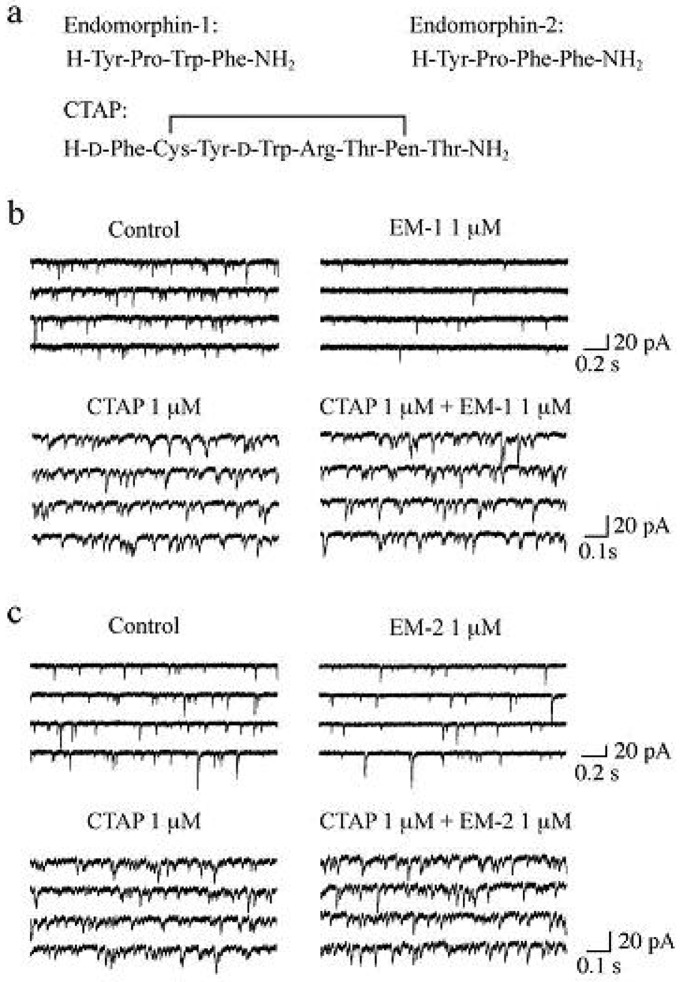
Actions of endomorphin-1 and endomorphin-2 (EM-1 and EM-2, respectively; each 1 μM) on spontaneous excitatory synaptic transmission in rat SG neurons in the absence and presence of a μ-opioid receptor antagonist CTAP (1 μM). (**a**) Amino-acid sequences of EM-1, EM-2 and CTAP; (**b, c**) Four consecutive traces of spontaneous EPSCs (sEPSCs) in the absence (left) and presence of EM-1 (right; **b**) or EM-2 (right; **c**) in Krebs solution without (upper) or with CTAP (lower). Note that EM-1(**b**) and EM-2 (**c**) reduce the frequency of sEPSC without a change in amplitude in a manner sensitive to CTAP. V_H_ = −70 mV. Modified from [90].

It has been demonstrated that the rat SG contains endogenous opioid peptides such as enkephalins [60,61], endomorphin-1 and endomorphin-2 (EM-1 and EM-2, respectively; see Figure 4a for their amino-acid sequences). EM-1 and EM-2, which were isolated from mammalian brain in 1997, possess high affinity and selectivity for the μ-opioid receptor as compared to the δ- and κ-opioid receptors [62-64]. There is much evidence showing that EM-1 and EM-2 play a pivotal role in inhibiting nociceptive transmission at the spinal cord level. Intrathecal administration of EM-1 and EM-2 produced antinociception in the tail-flick, paw-withdrawal, tail-pressure and flexor-reflex tests in adult rodents [62,65-72]. EM-1 and EM-2 like immunoreactive fibers have been shown to exist in the superficial laminae of the rat spinal cord [73-76] and in rat primary-afferent fibers [77,78]. EM-2 like immunoreactive fibers and terminals in the rat spinal dorsal horn have been demonstrated to originate from ipsilateral primary afferents and bilateral descending fibers from the nucleus tractus solitarii [79]. Furthermore, EM-2 like substances are released from the rat spinal dorsal horn in response to electrical stimulation applied to the dorsal root entry zone [80]. Axon terminals containing EM-2 like immunoreactivity make synapses with neurons immunostained for μ-opioid receptors in the rat spinal dorsal horn [81]. EM-1 and EM-2 are different by only one amino acid residue and thus exhibit similar antinociceptive potency at the spinal cord level in mice [65,69] and rats [67,72]. On the other hand, antinociceptive effects produced by them are distinct in the development of acute tolerance [65], in the extent [66,68] and in the duration [72] from each other.

Both EM-1 and EM-2 reduce primary-afferent C-fiber mediated responses while EM-1 but not EM-2 inhibits Aβ-fiber ones in rat dorsal horn neurons [82]. Such a distinction may not be unexpected, because there is a difference between EM-1 and EM-2 in the affinity to μ-opioid receptors, determined from binding experiments [62]. Gong *et al.* [83] have reported that the activation of K^+^ channels through cloned μ-opioid receptors differs in extent between EM-1 and EM-2 actions. Human μ-opioid receptors fused to G_i1_α or G_i2_α in transfected HEK 293 cells exhibited binding affinities which were different by 3-8-fold between EM-1 and EM-2 [84]. Behavioral studies have suggested that each of EM-1 and EM-2 may activate different μ-opioid receptor subtypes such as μ_1_ and μ_2_ [85], which are pharmacologically distinct, in the spinal dorsal horn [69,70], although there is no evidence for the presence of the μ-opioid receptor subtypes. A similar idea has been also applied to a difference between EM-1 and EM-2 in motivational effects and conditioned place preference responses, which are produced by their intracerebroventricular administrations [86,87]. There is a difference between EM-1 and EM-2 immunoreactivities in the distribution in the spinal dorsal horn in such that EM-2 exists at higher density than EM-1, suggesting a different role of EM-1 and EM-2 in spinal antinociception [74].

Spontaneous EPSCs (sEPSCs) were unaffected in frequency and amplitude by TTX (0.5 μM), indicating that the production of the sEPSCs was independent of the spontaneous activities of neurons presynaptic to SG neurons and thus sEPSCs were equivalent to mEPSCs. In the following, we examined the effects of EMs on sEPSCs, which were observed in Krebs solution without TTX. Superfusing EM-1 or EM-2 (each 1 μM) for 2 min resulted in a reduction in the occurrence of sEPSC, as shown in Figure 4b,c. A maximal reduction in sEPSC frequency was seen around 2 min after washout of EM-1 or EM-2; this magnitude was about 45%. On the other hand, sEPSC amplitude was unaffected by EM-1 or EM-2. Consistent with no change in sEPSC amplitude, EM-1 and EM-2 did not affect the peak amplitude of the response of SG neurons to AMPA (5 μM). Such a reduction in sEPSC frequency by EM-1 or EM-2 was not seen in the presence of CTAP (1 μM; see Figure 4a for its amino-acid sequence; Figure 4b,c). The sEPSC frequency reductions by EM-1 and EM-2 were not different in extent from each other. Similar reduction in sEPSC frequency by EM-1 in adult rat SG neurons has been reported by Yajiri and Huang [88].

The magnitudes (45%) of sEPSC frequency reductions produced by EM-1 and EM-2 (each 1 μM) were similar to a maximal reductive one (40%) of monosynaptic Aδ-fiber evoked EPSC amplitude [88] and also to those (about 40%) by EMs (1 μM) of short-latency evoked EPSCs in young rat SG neurons by stimulating the dorsal root entry zone [89]. Wu *et al.* [89] have reported that there is no difference between EM-1 and EM-2 in efficacy for reducing evoked EPSC amplitudes in young rat SG neurons, as seen for EMs-induced outward current (hyperpolarization) in postsynaptic membranes of adult rat SG neurons [90]. In conclusion, the difference in behaviorally-examined antinociceptive effects between EM-1 and EM-2 could not be attributed to a distinction in their pre- and postsynaptic effects on excitatory transmission in SG neurons, and may be explained by a difference in their enzymatic degradation [90]. This idea may be consistent with the observations that EM-1 required a longer pretreatment time than EM-2 before tolerance was observed [65] and that the duration of spinal antinociceptive effects was significantly longer for EM-1 than EM-2 [72]. A cellular mechanism for the presynaptic inhibition of evoked EPSC would be an inhibition of voltage-gated Ca^2+^ channels by opioids in nerve terminals, because EM-1 and EM-2 are reported to reduce Ca^2+^-channel currents in NGMO-251 cells expressing μ-opioid receptors [91].

### 2.3. Actions of Tramadol Metabolite M1

Tramadol is a clinically-used, orally-active drug, which is considered to act as an analgesic in the CNS [92]. The activation of μ-opioid receptors by tramadol has been revealed from the experimental results of a μ-opioid receptor binding of tramadol [93] and its [^35^S]GTP-γ-S binding stimulation [94]. Although tramadol is metabolized to various compounds including mono-*O*-demethyl tramadol (M1) via *N*- and *O*-demethylation in humans and animals [95], M1 is thought to be a therapeutically active drug as central analgesics [92]. M1 has the highest affinity for the cloned μ-opioid receptors among the metabolites of tramadol [94].

Under the condition where M1-induced postsynaptic current mediated by μ-opioid receptors [96] was inhibited, M1 (1 mM) superfused for 2 min reduced the frequency (by about 30%) but not amplitude of sEPSCs recorded at −70 mV; a response of SG neurons to bath-applied AMPA (10 μM) was unaffected by M1 (1 mM). This sEPSC frequency reduction persisted for at least 20 min after washout of M1. This inhibitory action of M1 was not seen in the presence of CTAP (1 μM). M1 (1 mM) also reduced the peak amplitudes of EPSCs which were monosynaptically evoked at −70 mV in SG neurons by stimulating primary-afferent Aδ-fiber and/or C-fiber in a spinal cord slice with an attached dorsal root. Each of the Aδ-fiber and C-fiber EPSC amplitude was reduced by M1 with a similar extent of 40–50%. This was so in a single neuron where both of the monosynaptic Aδ-fiber and C-fiber EPSCs were seen. It was concluded that M1 inhibits the quantal release of l-glutamate from the central terminals of primary-afferent neurons through the activation of μ-opioid receptors in the SG; this action is not distinct in extent between primary-afferent Aδ-fiber and C-fiber glutamatergic transmission [97].

## 3. Opioid Actions on the Peripheral Terminals of Primary-Afferent Neurons

There is much evidence supporting the idea that APs produced in the peripheral terminals of primary-afferent neurons in response to nociceptive stimuli given to the periphery are inhibited by an action of opioids. A shift in a voltage dependency of currents (I_h_s) through hyperpolarization-activated channels involved in neuronal excitability was produced by an adenylate cyclase activator forskolin, cyclic AMP analogue and one of membrane phospholipid metabolites, prostaglandin E_2_ (PGE_2_; which is involved in a sensitization of nociception in the peripheral terminal) in cultured guinea-pig nodose ganglion neurons. Such a change in I_h_ was reversed by opioids in a manner sensitive to naloxone [98]. DAMGO inhibited a modulation by PGE_2_ of TTX-resistant Na^+^ channels, which play a pivotal role in sensitization of nociceptors, in rat DRG neurons; this DAMGO action was inhibited by naloxone [99]. The TTX-resistant Na^+^ channels have been shown to be involved in the spontaneous and ectopic production of APs in chronic pain rat models [100] and also in visceral pain and referred hyperalgesia [36,37,101].

## 4. Opioid Actions on Peripheral Nerve Fibers

AP, which plays an important role in transmitting neuronal information in nerve fibers, is generally mediated by voltage-gated Na^+^ and K^+^ channels located in neuronal membranes. AP produced at a point of nerve fiber membrane electrotonically conducts to a nearby membrane where a depolarization is produced. This depolarization opens voltage-gated Na^+^ channels, resulting in Na^+^ influx, *i.e.,* inward current caused by the gradient of the electrochemical potential of Na^+^, and then in the production of AP. APs thus produced disappear owing to the inactivation of voltage-gated Na^+^ channels following their opening and also the activation of a delayed-rectifier type of voltage-gated K^+^ channels. When the opening and closing of Na^+^ and K^+^ channels occur in the longitudinal direction of a nerve fiber, a current flows on the extracellular surface of the fiber [102]. When many of nerve fibers contained in the nerve trunk are simultaneously stimulated by using an electrode put on the trunk, currents flowing on the surface of the fibers can be recorded as CAP by using a recording electrode put near the stimulating electrode. We examined the effect of opioids on fast-conducting frog sciatic nerve CAPs (conduction velocity: 30–40 m/s; this corresponds to that of A fibers) which are easy to be measured by using the air-gap method and well characterized in property.

### 4.1. Actions of Opioids on CAPs

**Figure 5:**
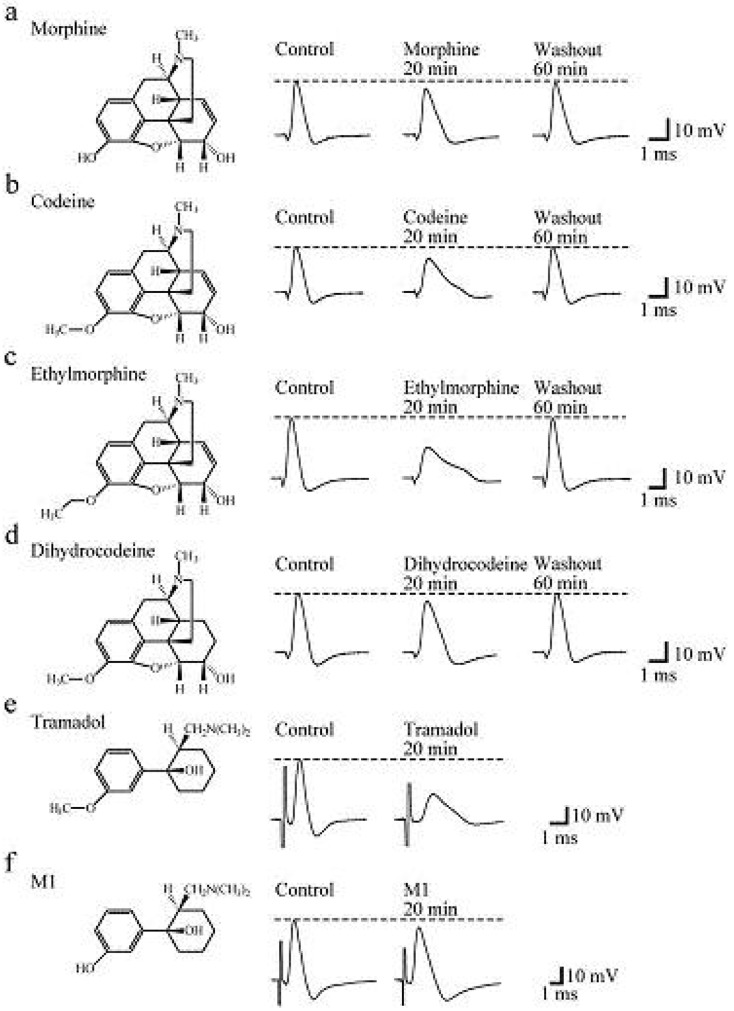
Opioids inhibit compound action potential (CAP) in the frog sciatic nerve, (**a-f**) Recordings of CAPs in the absence and presence of morphine (**a**), codeine (**b**), ethylmorphine (**c**), dihydrocodeine (**d**), tramadol (**e**) and M1 (**f**; each 5 mM). The most left-hand side inset in each of (**a-f**) shows the chemical structures of the opioids. Note that each of the opioids reduces CAP peak amplitude with a different extent. Modified from [103,105].

**Figure 6:**
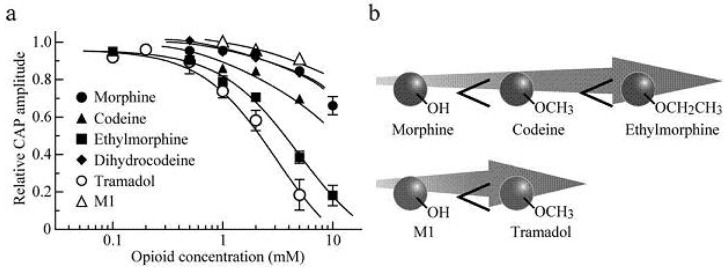
Summary of the effects of opioid-receptor agonists on CAPs in the frog sciatic nerve. (**a**) Comparison of concentration-response curves for CAP peak amplitude reductions among opioid-receptor agonists (morphine, codeine, ethylmorphine, dihydrocodeine, tramadol and M1). The curves for ethylmorphine and tramadol were drawn according to the Hill equation; the other curves by eye; (**b**) Schematic diagram showing a difference in the extent of CAP inhibition among morphine, codeine and ethylmorphine (upper) or between M1 and tramadol (lower). Modified from [103,105].

Soaking the sciatic nerve into morphine (5 mM)-containing Ringer solution reversibly reduced the peak amplitude of the CAP (Figure 5a). The morphine-induced reduction in CAP peak amplitude attained a steady effect within 20 min after the soaking. CAP amplitude reduction at a steady state increased in extent with an increase in morphine concentration. The concentration-response curve for the morphine-induced CAP amplitude reduction obtained from many nerve trunks is given in Figure 6a [103]. CAPs in the frog sciatic nerve were less sensitive to morphine than those in the guinea-pig and rabbit vagus nerves (formed by C fibers) in such that the peak amplitudes in the vagus nerves are reduced by 20–32% at 0.5 mM [42].

Codeine at a concentration of 5 mM also reduced CAP peak amplitude in a reversible manner (Figure 5b). Like morphine, codeine (5 mM) exhibited a steady effect of CAP amplitude reduction within 20 min after the soaking. The extent of the CAP peak amplitude reduction produced by codeine was enhanced with an increase in its concentration (Figure 6a; [103]). When compared at a concentration of 5 mM, codeine-induced reduction (about 30%) in CAP amplitude in the frog sciatic nerve was much smaller than that (about 70%) in the rat phrenic nerve (formed by A fibers; [104]), while there was not so a large difference in morphine action (about 10%).

Ethylmorphine at a concentration of 5 mM reversibly reduced CAP peak amplitude (Figure 5c). Figure 6a demonstrates the effects of ethylmorphine in a wide range of 0.1–10 mM on CAPs. The extent of the CAP peak amplitude reduction produced by ethylmorphine was enhanced with an increase in its concentration. Analysis based on the Hill equation showed that half-maximal inhibitory concentration (IC_50_) value for ethylmorphine is 4.6 mM with the Hill coefficient (n_H_) of 1.2 [103].

To more know a relationship between CAP inhibition by opioids and their chemical structures, we examined the effect of dihydrocodeine, where codeine was hydrogenated, on CAPs. Like other opioids, dihydrocodeine at a concentration of 5 mM reversibly reduced CAP peak amplitude (Figure 5d). The extent of this reduction was smaller than those of codeine and ethylmorphine while being almost comparable to that of morphine. Figure 6a demonstrates the effects of dihydrocodeine in a wide range of 0.5–5 mM on CAPs. The extent of the CAP peak amplitude reduction produced by dihydrocodeine was enhanced with an increase in its concentration [103].

In order to know whether the opioid-induced reduction of CAP peak amplitude is mediated by opioid receptors, we examined the effects of naloxone on the CAP amplitude reductions produced by opioids. The CAP peak reductions produced by morphine (10 mM), codeine (5 mM), ethylmorphine (2 mM) and dihydrocodeine (5 mM) in the presence of naloxone (0.01 mM) were not significantly different in extent from those in the absence of this opioid-receptor antagonist [103]. Thus, the opioid actions in frog sciatic nerve fibers were resistant to naloxone, although naloxone-sensitive CAP inhibition has been reported in mammalian and amphibian peripheral nerve fibers [42,46]. Our result is consistent with previous reports [39,40,104] showing naloxone-insensitive CAP amplitude reduction and nerve conduction inhibition produced by opioids.

When the inhibitory action of ethylmorphine was compared with those of local anesthetics, the IC_50_ value for this opioid was larger by about six-fold than those of lidocaine and cocaine (0.74 mM and 0.80 mM, respectively; [103,105]).

### 4.2. Actions of Tramadol and M1 on CAPs

Soaking the sciatic nerve into tramadol (1 mM)-containing solution resulted in reducing the peak amplitude of the CAP (Figure 5e). The tramadol-induced reduction in CAP peak amplitude attained a steady effect within 20 min after the soaking. At least 1 h after soaking the sciatic nerve into tramadol-free solution, the CAP amplitude did not recover to control level. The CAP peak amplitude reduction produced by tramadol was enhanced in extent with an increase in its concentration. Figure 6a demonstrates the effect of tramadol in a wide concentration range of 0.2 to 5 mM on CAPs. Analysis based on the Hill equation showed that IC_50_ value for tramadol is 2.3 mM with the n_H_ of 1.7 [105]. A similar CAP amplitude reduction produced by tramadol has been obtained by applying the sucrose-gap method to frog [41] and rat sciatic nerves [106,107]. The tramadol action in the rat sciatic nerve has been suggested to be larger for fast-conducting than slow-conducting CAPs [108]. IC_50_ value (2.3 mM) for tramadol in our study was smaller by about three-fold than that (6.6 mM) obtained previously for the frog sciatic nerve [41]. Since tramadol exhibits a high affinity for opioid receptors [109], we next investigated whether the tramadol effect is mediated by opioid receptors. Pretreatment for 20 min of sciatic nerves with naloxone (0.01 mM) did not affect the tramadol-induced inhibition of CAP [105]. Consistent with this observation, Tsai *et al.* [110] have reported that a reduction in spinal somatosensory evoked potentials following the application of tramadol on rat sciatic nerves *in vivo* is resistant to naloxone. Moreover, a μ-opioid receptor agonist DAMGO at 0.001 mM, a concentration maximally activating μ-opioid receptors in rat SG neurons [90], did not affect CAPs.

In order to know further whether the tramadol-induced reduction in CAP peak amplitude is related to μ-opioid receptor activation, we examined the effect on sciatic nerve CAPs of M1, which is similar in chemical structure to tramadol (see Figures 5e,f) while having a higher affinity for the receptors than tramadol does [95]. CAPs were not affected by soaking the sciatic nerve into Ringer solution containing M1 at a concentration of 1 or 2 mM for 20 min [105]. M1 at a higher concentration such as 5 mM reduced CAP peak amplitude by about 9% (Figure 5f and Figure 6a).

No involvement of G-protein coupled opioid receptors in CAP inhibition by various opioids is consistent with the fact that our preparation is the dissected sciatic nerve devoid from the neuronal cell body and the neuronal terminals.

The CAP peak amplitude reduction produced by tramadol in frog sciatic nerves may provide a basis for a local anesthetic effect following its intradermal injection in patients [111-113]. Consistent with the fact that the IC_50_ value for tramadol in reducing CAP peak amplitude is larger by 3.1-fold than that of lidocaine [105], a sensory block at the intradermal injection site of 5% tramadol was similar to that of 1% lidocaine [111].

Since alcohols, anticonvulsants, barbiturates and narcotics block AP conduction in peripheral nerve fibers [114], the effects of opioids on CAPs in our study may be due to nonspecific interactions with membrane bilayers and also ion channels such as voltage-gated Na^+^ and K^+^ channels [115]. In support of the latter idea, Wagner *et al.* [116] have reported that an opioid meperidine, which is used for AP conduction blockade and thus analgesia, reduces voltage-gated Na^+^-channel current amplitudes. Tsai *et al.* [117] have demonstrated that tramadol attenuates the current amplitude of delayed-rectifier K^+^ channels (Kv3.1a type) expressed in NG 108-15 cells. Tramadol also inhibits heterologously expressed neuronal voltage-gated Na^+^ channels (Nav1.2 type; [118]). Hu and Rubly [119] have reported in single myelinated nerve fibers isolated from the frog sciatic nerve that morphine depresses steady-state K^+^ currents and peak Na^+^ currents, resulting in the prolongation of APs. Frazier *et al.* [120] have demonstrated that intracellularly-applied morphine reduces voltage-gated Na^+^- and K^+^-channel current amplitudes in squid giant axons.

Figure 6b demonstrates a schematic illustration of a difference in the extent of conduction inhibition among morphine, codeine and ethylmorohine and also between M1 and tramadol. Although morphine, codeine and ethylmorphine are distinct in chemical structure in terms of only a group attached to a benzene ring in such that they have -OH, -OCH_3_ and -OCH_2_CH_3_, respectively, this reduction enhanced in extent with an increase in the number of -CH_2_. Alternatively, tramadol having -OCH_3_ in a benzene ring reduced CAP peak amplitudes more effectively than M1 which is different from tramadol only in terms of the presence of -OH in the ring. This is so, although the chemical structures of morphine, codeine and ethylmorphine are quite different from those of tramadol and M1 (Figure 5). Since the increase in the number of -CH_2_ is expected to enhance lipophilicity of opioids, it is suggested that lipophilic opioid-channel interactions may play an important role in nerve conduction block, as shown for local anesthetics [121,122]. In support of this idea, the potency in inhibiting CAPs in the rat sciatic nerve was in the order of cocaine < cocaethylene (where the methyl ester group of cocaine was replaced with an ethyl ester group) < isopropylcocaine (where its methyl ester group was replaced with an isopropyl ester group; [123]). It would be of interest to note that the sequence of the affinity of opioids for μ-opioid receptors is morphine > codeine > ethylmorphine [124], the order of which is reversed to one for CAP inhibition. If the opioid-induced inhibition of CAPs is mediated by μ-opioid receptors, CAP inhibition sequence will be expected to be morphine > codeine > ethylmorphine. However, this is not the case, a result being consistent with our idea that the opioids-induced CAP inhibition in the frog sciatic nerve is not mediated by opioid receptors. Such a difference in chemical structure could serve as information to know molecular mechanisms for the inhibition of AP conduction by opioids.

The inhibition of AP conduction by opioids in large diameter (A-type) fibers might contribute to local anesthesia following peripheral perineural injections of opioids which are expected to result in a direct action of opioids at high doses on peripheral nerves, such as intradermal injection. Although sensory information is transmitted by not only fast- but also slow-conducting fibers in sciatic nerves, the present study does not examine the effects of opioids on slow-conducting APs in small caliber and unmyelinated axons which are involved in peripheral analgesia [125]. In order to more firmly establish a clinical significance of CAP amplitude reduction produced by opioids, their effects on slow-conducting CAPs such as TTX-resistant ones [126] remain to be examined. Moreover, it remains to be examined whether the structure-function relationship of opioids is applied to slow-conducting APs. Since codeine is metabolized to morphine *via O*-demethylation in animals and humans [29,127,128], peripherally-applied codeine might have a similar effect to that of morphine.

It remains to be examined how APs in primary-afferent fibers are affected by opioids in order to clearly know their actions on sensory transmission, because the sciatic nerve contains not only afferent (sensory) but also efferent (motor) fibers.

## 5. Conclusions

Opioids inhibit nociceptive transmission in primary-afferent neurons with opioid-receptor activation. Opioid receptors located in the central and peripheral terminals of primary-afferent neurons are involved in a decrease in the release of l-glutamate onto spinal SG neurons and an inhibition of the production of APs in response to nociceptive stimuli given to the periphery, respectively (Figure 1). These opioid-receptor activation results in antinocicetion, *i.e.,* analgesia. Opioids at concentrations much higher than required to activate opioid receptors inhibit AP conduction in primary-afferent fibers (see Figure 1), possibly by inhibiting voltage-gated Na^+^ channels, and thus exhibit an anesthetic action. Comparison in the extent of conduction inhibition among opioids revealed that a chemical structure of opioids plays an important role in inhibiting nerve conduction. Although this inhibitory effect of ethylmorphine is smaller than that of lidocaine, the structure-activity relationship revealed in this study may serve to develop an opioid which has a local anesthetic effect more effective than lidocaine.
